# Measurements of spontaneous CFTR-mediated ion transport without acute channel activation in airway epithelial cultures after modulator exposure

**DOI:** 10.1038/s41598-021-02044-1

**Published:** 2021-11-19

**Authors:** Heidi J. Nick, Pamela L. Zeitlin, Sangya Yadav, Preston E. Bratcher

**Affiliations:** 1 Department of Pediatrics, National Jewish Health, 1400 Jackson Street, Denver, CO 80206 USA; 2 Department of Pediatrics, Anschutz Medical Center, University of Colorado Denver, Aurora, CO USA

**Keywords:** Electrophysiology, Respiration, Biologics

## Abstract

Quantitation of CFTR function in vitro is commonly performed by acutely stimulating then inhibiting ion transport through CFTR and measuring the resulting changes in transepithelial voltage (V_te_) and current (I_SC_). While this technique is suitable for measuring the maximum functional capacity of CFTR, it may not provide an accurate estimate of in vivo CFTR activity. To test if CFTR-mediated ion transport could be measured in the absence of acute CFTR stimulation, primary airway epithelia were analyzed in an Ussing chamber with treatment of amiloride followed by CFTR(inh)-172 without acute activation of CFTR. Non-CF epithelia demonstrated a decrease in V_te_ and I_SC_ following exposure to CFTR(inh)-172 and in the absence of forskolin/IBMX (F/I); this decrease is interpreted as a measure of spontaneous CFTR activity present in these epithelia. In F508del/F508del CFTR epithelia, F/I-induced changes in V_te_ and I_SC_ were ~ fourfold increased after treatment with VX-809/VX-770, while the magnitude of spontaneous CFTR activities were only ~ 1.6-fold increased after VX-809/VX-770 treatment. Method-dependent discrepancies in the responses of other CF epithelia to modulator treatments were observed. These results serve as a proof of concept for the analysis of CFTR modulator responses in vitro in the absence of acute CFTR activation. Future studies will determine the usefulness of this approach in the development of novel CFTR modulator therapies.

## Introduction

The treatment of cystic fibrosis (CF) has been revolutionized by precision medicine approaches. This is evident in the development and use of cystic fibrosis transmembrane conductance regulator (CFTR) modulators^1^. These novel compounds target the dysfunctional protein responsible for CF disease manifestations and enhance CFTR-mediated chloride transport in a mutation-specific manner. Clinically, these compounds have proven to be very effective in the treatment of CF.

Analysis of ion transport across an epithelium is a valuable tool for the measurement of cellular responses to treatment with CFTR modulating compounds. Investigations of Fischer rat thyroid (FRT) cells expressing mutant CFTRs and the drug ivacaftor served as evidence for the FDA-approval of this drug for individuals with CFTR mutations that were responsive in vitro^2,3^. Clinical effectiveness of ivacaftor in individuals with at least one gating mutation confirmed the in vitro findings^4,5^, demonstrating the overwhelming potential of in vitro studies for the discovery and development of efficacious drug treatments for individuals with CF. More recently, the FRT cell model was utilized in testing the combination modulators tezacaftor/ivacaftor and elexecaftor/tezacaftor/ivacaftor, with subsequent FDA approval for use to treat individuals with mutations for which increases in function were observed^6,7^. As next generation modulators continue to emerge, the analysis of efficacy in meaningful models, especially in primary airway epithelial cultures, will continue to be of importance.

A standard protocol for acute ion channel manipulations in cultures mounted in an Ussing chamber consists of inhibiting sodium transport by application of amiloride to the apical chamber, followed by activation of CFTR-mediated chloride transport using forskolin and/or 3-isobutyl-1-methylxanthine (IBMX) applied to both the apical and basal chambers, then inhibition of CFTR using CFTR(inh)-172 applied to the apical chamber. Forskolin is an activator of adenylate cyclase and IBMX is an inhibitor of phosphodiesterase; both compounds serve to increase intracellular concentrations of 3′,5′-cyclic adenosine monophosphate (cAMP), thereby stimulating transport through the CFTR channel.

Given that forskolin and IBMX are not utilized for the treatment of CF in vivo either alone or in CFTR modulator combination therapies, applying these compounds acutely during electrophysiological analyses of CFTR modulator-treated cultures may result in the misestimation of modulator effectiveness. The aim of the present study was to explore the potential of assessing modulator responses in vitro by examining changes in levels of CFTR activity present in epithelial cultures in the absence of forskolin and IBMX.

## Results

### Nasal airway epithelia derived from individuals without CF demonstrate spontaneous CFTR activity

**Figure 1:**
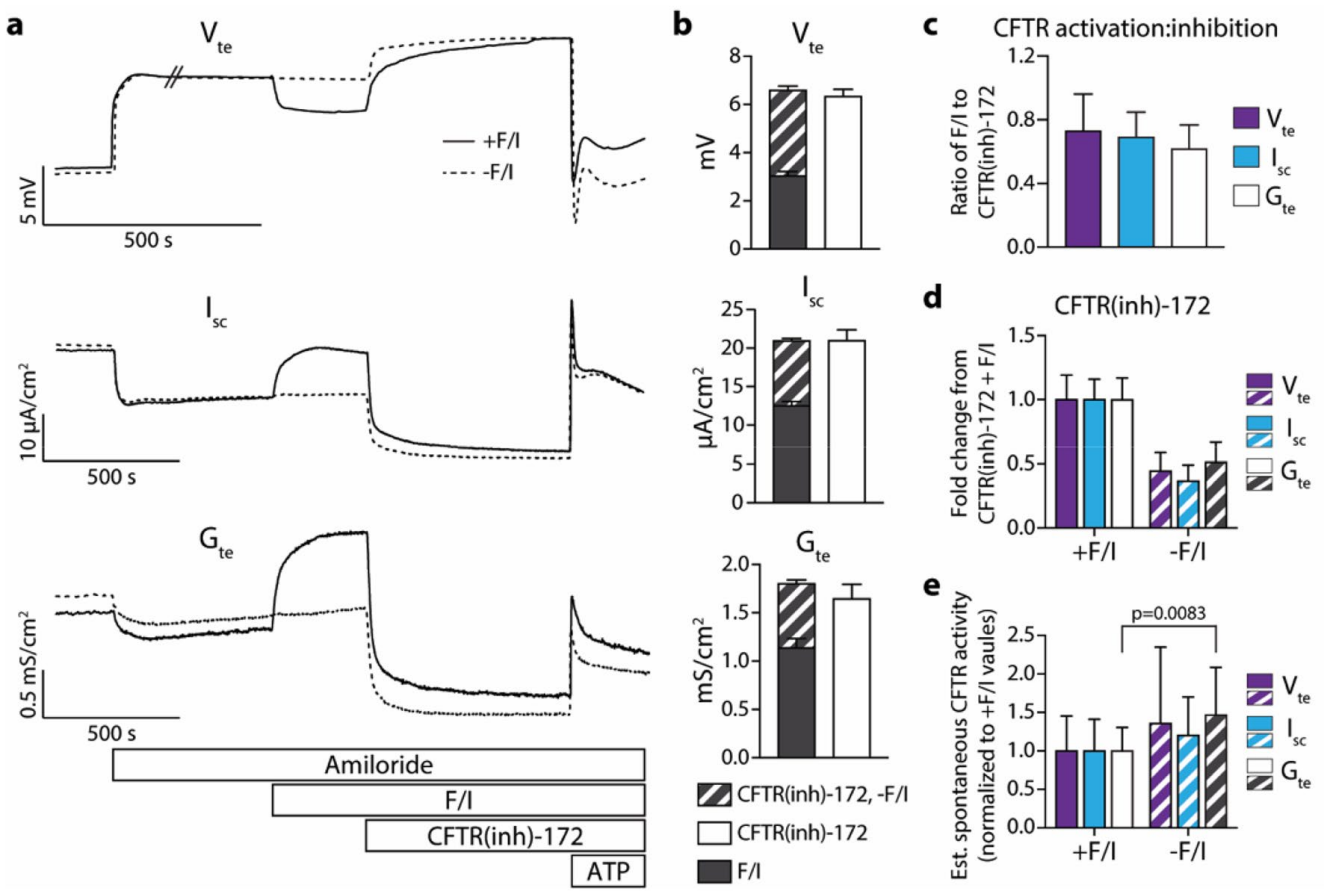
Non-CF nasal epithelia possess spontaneously active CFTR. Ion transport in non-CF nasal epithelia was analyzed in an Ussing chamber under open-circuit conditions with intermittent short-circuiting and voltage pulsing. (**a**) Representative tracings of transepithelial potential difference/voltage (V_te_), short-circuit current (I_SC_), and transepithelial conductance (G_te_) from epithelia analyzed in symmetrical Ringer’s solutions with sequential treatments of amiloride (100 µM), forskolin (20 µM)/IBMX (100 µM) (F/I) for acutely activated epithelia only, CFTR(inh)-172 (10 µM) and ATP (100 µM). (**b**) Quantification of responses to treatments are shown for the representative epithelia depicted in Panel (**a**). (**c**) For acutely activated epithelia, the ratio of the change in response to F/I to the change in response to the subsequent CFTR(inh)-172 treatment were calculated. Values shown are absolute. (**d**) Responses to CFTR(inh)-172 were normalized to the values obtained in epithelia treated with F/I prior to inhibition. (**e**) Spontaneous CFTR activity was estimated by subtracting values prior to F/I addition from values after CFTR(inh)-172 treatment. Changes in values from these estimates and from experiments without the use of F/I were normalized to the estimated values. n = 3 donors with 4 replicates per donor/condition.

In order to determine the amount of spontaneous CFTR activity present in the airway epithelia utilized in the present study, non-CF cultures were mounted in an Ussing chamber and acutely exposed to amiloride followed directly by CFTR(inh)-172. While amiloride is not utilized for the treatment of CF, inhibiting the epithelial sodium channel allows for accurate measurement of spontaneous and stimulated chloride transport^8–10^. All epithelia tested displayed a decrease in transepithelial potential difference/voltage (V_te_), short-circuit current (I_SC_), and transepithelial conductance (G_te_) upon treatment with CFTR(inh)-172 (Fig. 1), suggesting spontaneous CFTR activity is present in these cultures in the absence of induced CFTR activation. While it is possible that ion transport inhibited by CFTR(inh)-172 may be due at least in part to channels other than CFTR^11^, it is demonstrated below using CF epithelia that the magnitude of the observed inhibition is dependent on CFTR. Replicate cultures were treated in parallel with the standard sequence of amiloride, forskolin/IBMX (F/I), and CFTR(inh)-172. In F/I-treated epithelia, the responses to CFTR(inh)-172 were greater in magnitude than the responses to F/I [Fig. 1b (solid bars), c], and the difference in this magnitude could be attributed to spontaneous CFTR activity (Fig. 1b, striped bars). The responses to CFTR(inh)-172 in the absence of F/I were around half of the responses observed to CFTR(inh)-172 in the presence of F/I (Fig. 1d); this effect is due to the inhibition of both F/I-activated and spontaneously active CFTR-mediated currents by CFTR(inh)-172. Estimates of the levels of spontaneous CFTR activity can be made using measurements taken during analyses with F/I by subtracting values immediately before the addition of F/I from the values obtained after CFTR inhibition. However, these estimates vary slightly from the values obtained in the absence of F/I, indicating that F/I may induce responses likely through increased intracellular cAMP that are not affected by CFTR(inh)-172 (Fig. 1e). Overall, these results demonstrate that CFTR in cultures of primary non-CF nasal airway epithelia are actively transporting ions in the absence of exogenous activation and that this activity can be analyzed by measuring inhibition of CFTR.

### Modulation of F508del/F508del CFTR airway epithelia with VX-809/VX-770 increases F/I-induced changes in ion transport to a larger degree than spontaneous CFTR activity

**Figure 2:**
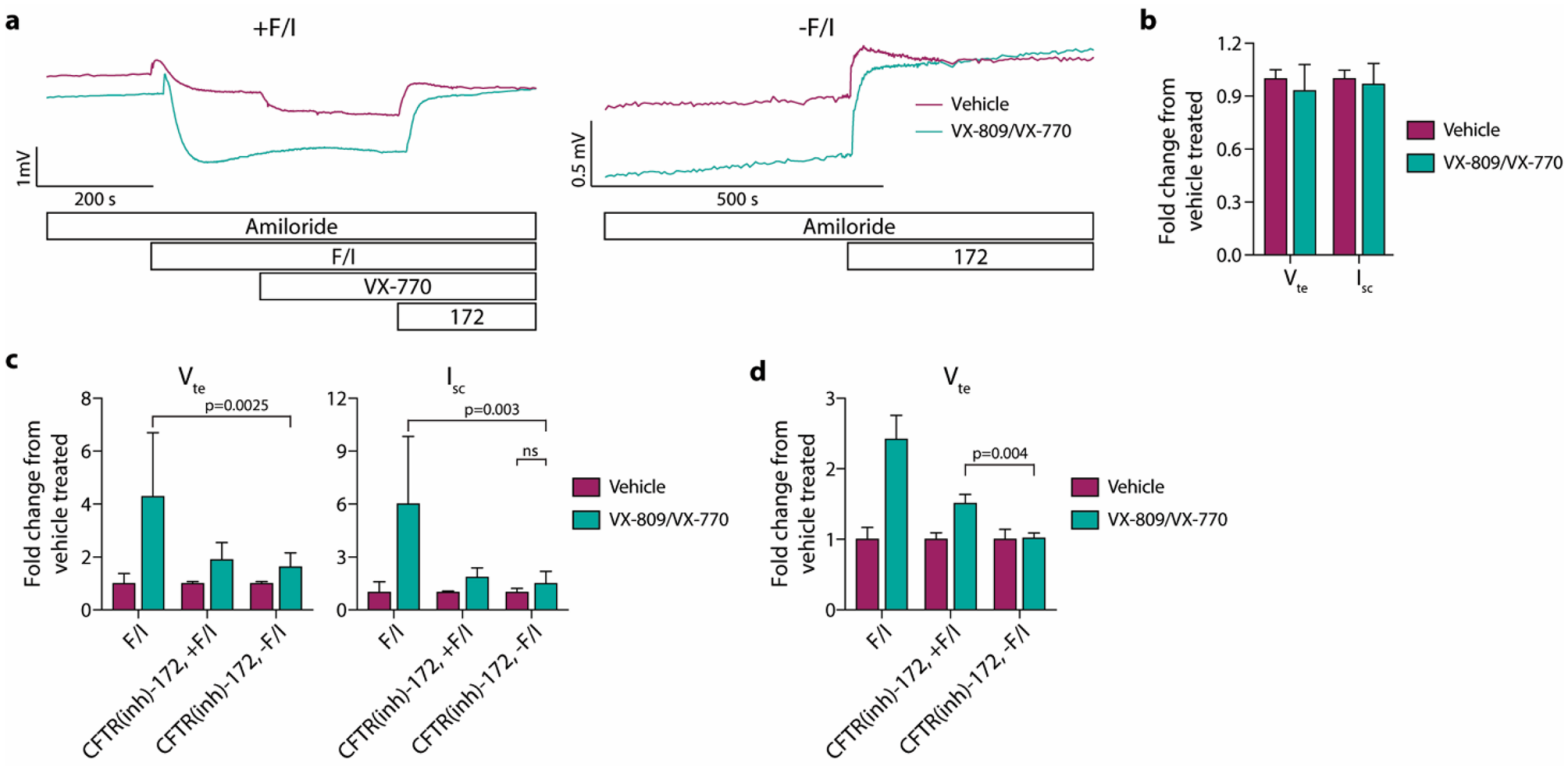
F508del/F508del CFTR nasal epithelia demonstrate low levels of spontaneously active CFTR-mediated ion transport that are slightly increased following 24 h VX-809/VX-770 treatment. Epithelia were analyzed in an Ussing chamber using the same methods as for non-CF epithelia, with the addition of acute 1 µM VX-770 between forskolin/IBMX (F/I) and CFTR(inh)-172 treatments for Panels (**a**–**c**). (**a**) Representative tracings of epithelia analyzed after treatment with vehicle vs. VX-809/VX-770. (**b**) Baseline values of transepithelial potential difference/voltage (V_te_) and short-circuit current (I_SC_) were normalized to the vehicle values in donor-matched replicate epithelia. (**c**) Responses to F/I or CFTR(inh)-172 [in the presence (+ F/I) or absence (-F/I) of F/I] treatments were normalized to the change in values in vehicle-treated epithelia. (**d**) Epithelia from a single donor were analyzed without acute VX-770 treatment (n = 3 replicates per condition). For Panels (**b**, **c**), n = 3 donors with 3 replicates per donor/condition. For data shown in Panel c, VX-809/VX-770 treatment resulted in significant increases in F/I and CFTR(inh)-172 for all cases (*p* < 0.003) with the exception of spontaneous CFTR-mediated changes in I_sc_.

The impact of correction and potentiation of F508del CFTR on spontaneous CFTR activity was analyzed in nasal airway epithelia derived from individuals with CF that were homozygous for the F508del CFTR mutation. 24-h treatment with the CFTR modulator combination of VX-809/VX-770 resulted in the expected increase in the responses to F/I [Fig. 2a (tracings on the left), c], while baseline V_te_ and I_SC_ values were not significantly changed (Fig. 2b). VX-770 was included in the 24-h modulator exposure in order to replicate in vivo therapy. For epithelia treated with F/I in the Ussing chamber, the F/I treatment was followed by acute VX-770 treatment (Fig. 2a); F508del CFTR in the vehicle-treated epithelia was potentiated, while there was no response in the VX-809/VX-770 exposed epithelia, demonstrating saturation of the F508del CFTR with VX-770. In parallel, replicate epithelial were analyzed without F/I [Fig. 2a (tracings on the right), c] and exhibited small responses to CFTR(inh)-172 which was increased after VX-809/VX-770 exposure. The responses to CFTR(inh)-172 after F/I and VX-770 treatments shown in Panel c were not deemed to be valuable measurements, as the acute VX-770 response resulted in an increase in the subsequent CFTR(inh)-172 response in the vehicle-treated epithelia; this, in turn, causes the fold increase in the values obtained for the VX-809/VX-770-exposed epithelia to decrease. This effect was demonstrated in epithelia analyzed with acute F/I treatment but without acute VX-770 (Fig. 2d); with acute VX-770 treatment, the CFTR(inh)-172 responses in modulator-treated epithelia are similar in the presence and absence of F/I, but without acute VX-770, the CFTR(inh)-172 response in the presence of F/I is greater than in the absence of F/I. Importantly, the effect of CFTR modulation was greater if measured as the response to F/I as compared to if measuring the change in spontaneous CFTR activity using the response to CFTR(inh)-172 in the absence of F/I (Fig. 1c). Conductance values obtained during the experiments depicted in Fig. 2 and all subsequent figures are provided in Supplementary Fig. 2.

### Chronic exposure of airway epithelia expressing G551D and R117H CFTRs to VX-770 increases spontaneous CFTR activity

**Figure 3:**
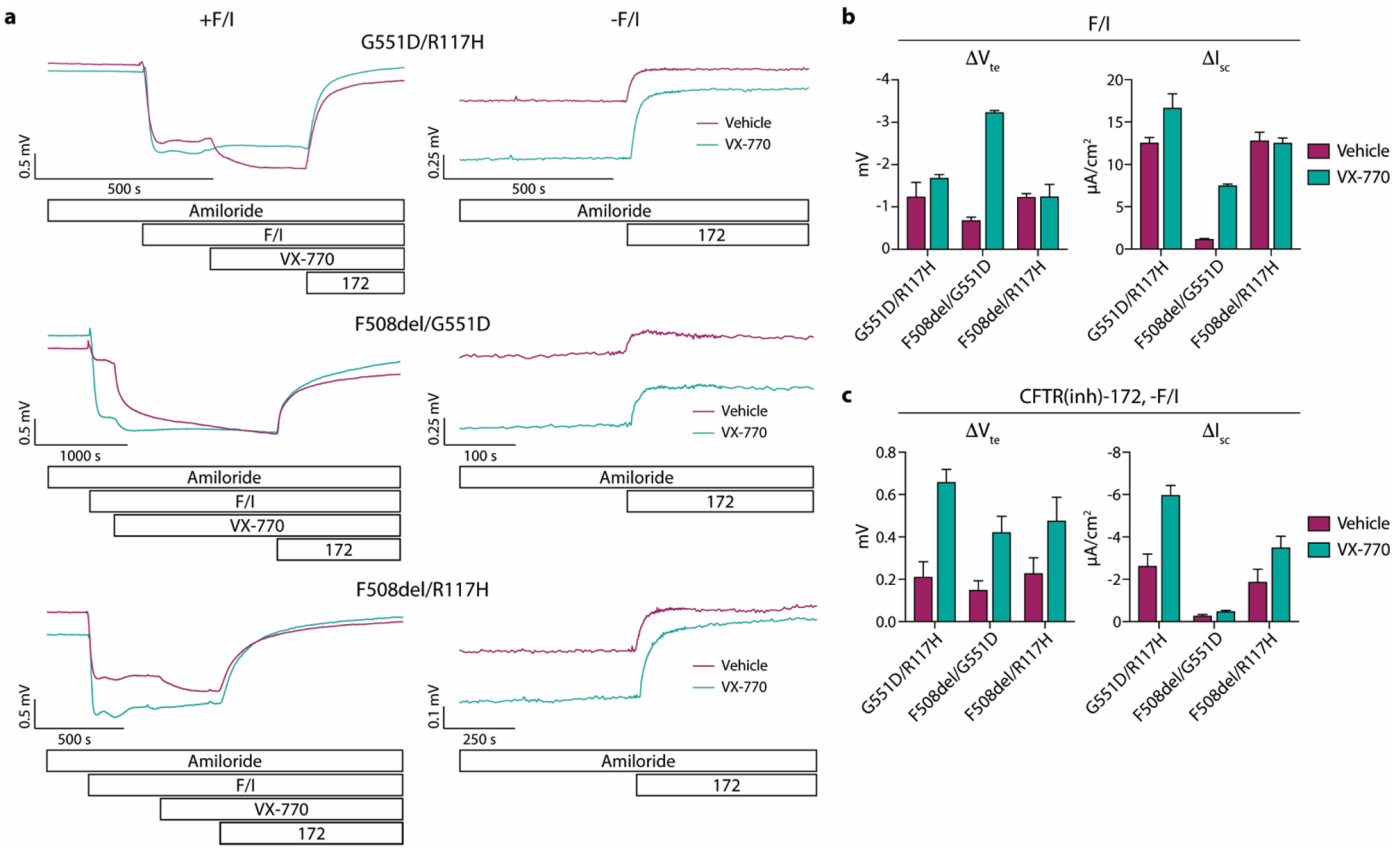
Nasal epithelial cell cultures derived from CF individuals expressing gating mutations in CFTR demonstrate responses in spontaneous CFTR activity after 24 h VX-770 treatment. Epithelia were analyzed in an Ussing chamber using the same methods listed in Figs. 1 and 2. (**a**) Representative tracings of epithelia analyzed after treatment with vehicle vs. VX-770 are shown; CFTR genotypes are listed above tracings. (**b**) Responses to forskolin/IBMX (F/I) were quantified. (**c**) Responses to CFTR(inh)-172 in the absence of F/I were quantified. n = 1 donor per genotype with 3–4 replicates per condition.

The experiments described above for F508del/F508del CFTR epithelia were repeated in epithelia derived from individuals with the CFTR genotypes G551D/R117H, F508del/G551D, and F508del/R117H (Fig. 3). These epithelial were treated for 24-h with VX-770 alone, as VX-770 has been shown to rectify the CFTR gating defect imparted by the G551D and R117H mutations^3,12,13^. Increases in the F/I-induced currents were observed in VX-770-treated epithelia with G551D CFTR mutations (Fig. 3b). Importantly, the spontaneous CFTR activity levels were increased for all three epithelia after treatment with VX-770, including the F508del/R117H epithelia which had no increase in the F/I-induced currents (Fig. 3c). When comparing the magnitudes of the effect of VX-770 measured using F/I-induced responses to these effects on spontaneous CFTR activities, significant differences between these measures of drug response were evident for epithelia of all genotypes (for G551D/R117H, *p* = 0.0001 for ∆V_te_ and *p* = 0.0012 for ∆I_sc_; for F508del/G551D, *p* < 0.0001 for ∆V_te_ and *p* < 0.0001 for ∆I_sc_; for F508del/R117H, *p* = 0.0434 for ∆V_te_ and *p* = 0.0097 for ∆I_sc_).

### VX-445/VX-661/VX-770 treatment of F508del/F508del CFTR airway epithelia dramatically increases spontaneous CFTR activity

**Figure 4:**
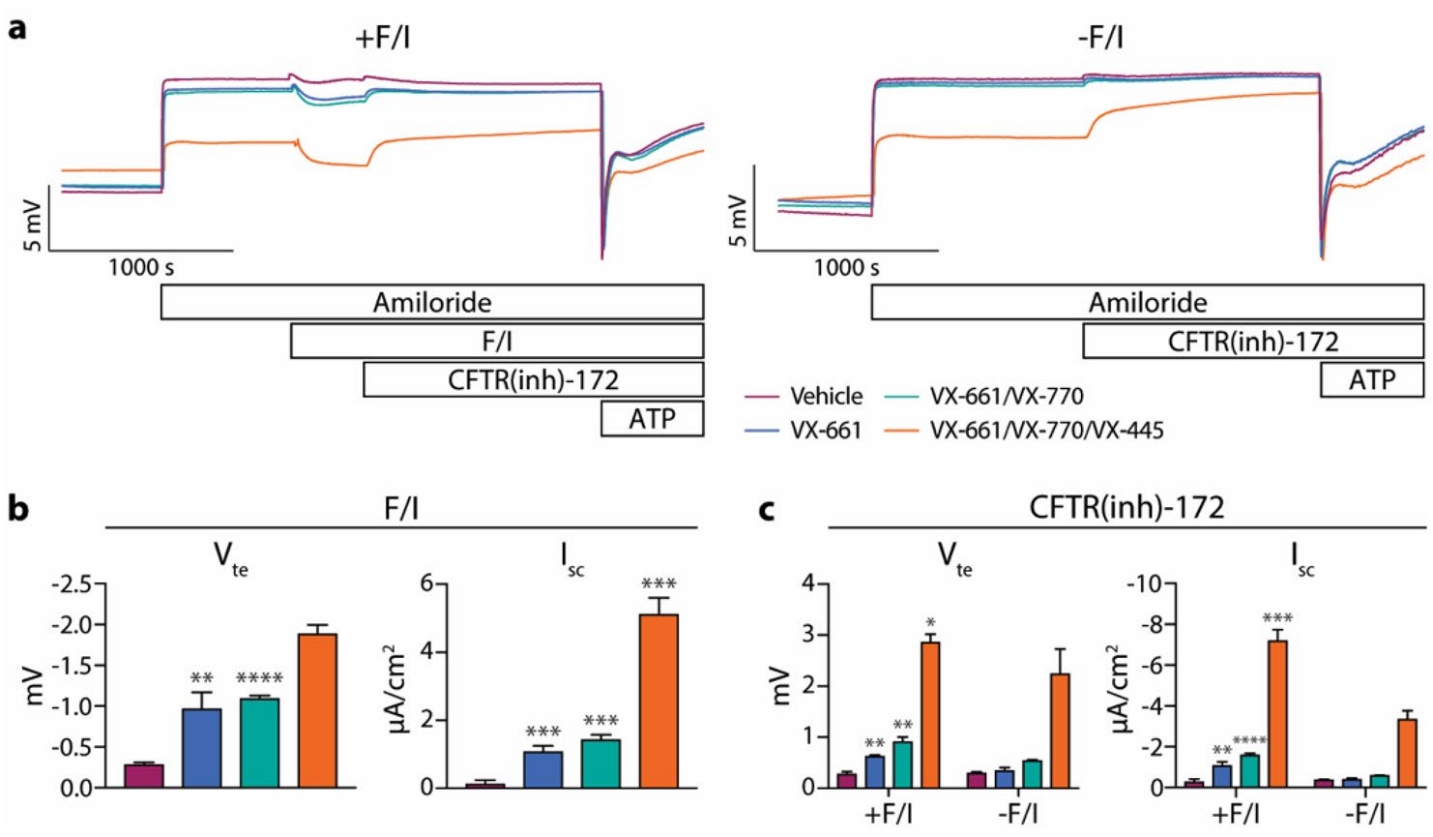
F508del/F508del CFTR nasal epithelia demonstrate dramatic increases in spontaneous CFTR activity after 24-h VX-445/VX-661/VX-770 treatment. Epithelia were analyzed in an Ussing chamber using the same methods listed in Fig. 1. (**a**) Representative tracings of epithelia analyzed after drug treatments. Responses to forskolin/IBMX (F/I) (**b**) and CFTR(inh)-172 in the presence (+ F/I) and absence (− F/I) of F/I (**c**) were quantified. In Panels (**b**) and (**c**), modulator treatments resulted in significant increases in F/I- and CFTR(inh)-172-mediated changes in transepithelial potential difference/voltage (V_te_) and short-circuit current (I_sc_) over vehicle-treated epithelia for all cases (*p* ≤ 0.0051) with the exception of spontaneous CFTR-mediated changes in epithelia treated with VX-661 alone; asterisks denote differences in the fold increase over vehicle-treated epithelia for the indicated group as compared to the spontaneous CFTR-mediated change for the same group and parameter. n = 3 replicates per condition.

Using the same experimental setup described above, the responses of F508del/F508del CFTR epithelia to three different modulator combinations were measured (Fig. 4). All modulator treatments resulted in increased F/I-induced responses [Fig. 4a (tracings on the left), b]. However, VX-661 treatment did not cause any changes in the spontaneous CFTR activity in these epithelia, while VX-661/VX-770 (similarly to VX-809/VX-770 above) resulted in only slight increases in the spontaneous CFTR activity (Fig. 4c). The effects of exposure to TRIKAFTA^®^ (VX-445/VX-661/VX-770) or components thereof in F508del CFTR-expressing cells have previously been described^14–19^, including the demonstration of an increase in spontaneous CFTR activity as estimated in Fig. 1e as well as an increase in post-amiloride basal currents^14,16,17,19^. In the present study, VX-445/VX-661/VX-770 treatment resulted in much greater increases in the spontaneous CFTR activity present in these epithelia and, in addition, significantly reduced the baseline V_te_, increased baseline G_te_ (Fig. S1a), and reduced the changes in V_te_ and I_sc_ in response to amiloride (Fig. S1b). Estimates of spontaneous CFTR activity taken from epithelia treated with F/I (as in Fig. 1e) were significantly different from the spontaneous CFTR activity values obtained in the absence of F/I, and the estimated values were consistently smaller in magnitude (Fig. S1c).

### Culture media composition influences the levels of spontaneous CFTR activity in airway epithelial cultures

**Figure 5:**
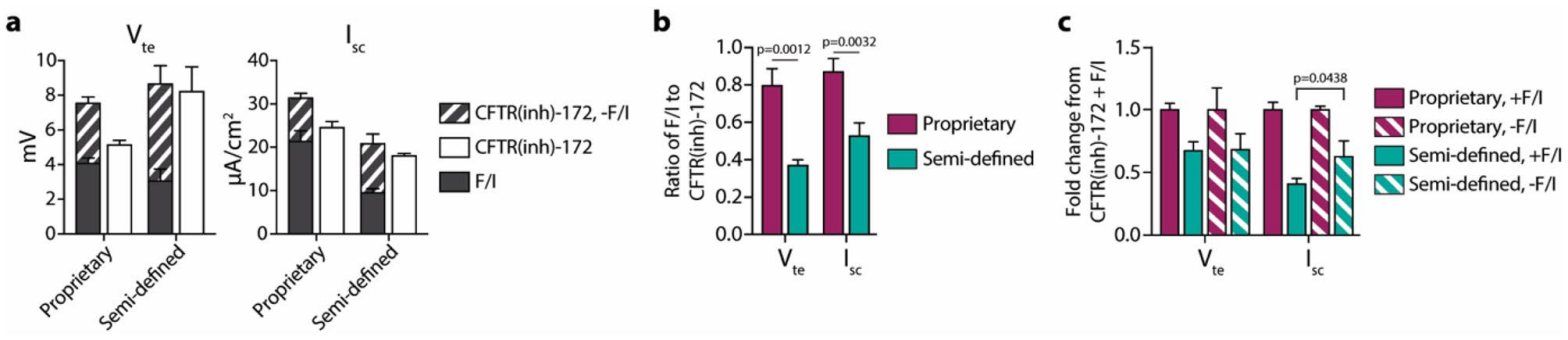
Media utilized for culture maintenance affects levels of spontaneous CFTR activity in non-CF nasal epithelia. Prior to analysis, epithelia were cultured for 96-h in either a proprietary media or a semi-defined media (see “Methods” section for additional details). Epithelia were analyzed in an Ussing chamber using the same methods listed in Fig. 1. (**a**) Responses to forskolin/IBMX (F/I) and CFTR(inh)-172 in the presence or absence of F/I were quantified. Significant differences due to media were detected for F/I responses [*p* = 0.0015 for short-circuit current (I_sc_)], post-F/I CFTR(inh)-172 responses [*p* = 0.0205 for transepithelial potential difference/voltage (V_te_), *p* = 0.0017 for I_sc_], and spontaneous CFTR activity (*p* = 0.0277 for V_te_). (**b**) For acutely activated epithelia, the ratio of the change in response to F/I to the change in response to the following CFTR(inh)-172 treatment were calculated. Values shown are absolute. (**c**) Responses to 172 were normalized to the values obtained in epithelia treated with F/I prior to inhibition. n = 1 donor with 3 replicates per condition.

Given the use of a proprietary culture media for the maintenance of the airway epithelia in the above experiments, it was necessary to analyze epithelia maintained in a semi-defined culture media in order to determine if components of the proprietary media were responsible for the spontaneous CFTR activities observed above. After three weeks at air–liquid interface, epithelia cultured using the same conditions utilized for all other epithelia were switched to a semi-defined media for 96 h prior to analysis in an Ussing chamber. Epithelia incubated in the semi-defined media had decreased responses to F/I (Fig. 5a) and ratios of F/I response to subsequent CFTR(inh)-172 response (Fig. 5b) as compared to replicate epithelia continuously incubated in the proprietary media. These differences were due to higher levels of spontaneous CFTR activity in the epithelia incubated in the semi-defined media [Fig. 5a (striped bars)], which is also demonstrated in the larger CFTR(inh)-172 response in the absence of F/I when normalized to the CFTR(inh)-172 response in the presence of F/I (Fig. 5c). Interestingly, while the V_te_ responses to CFTR inhibition after F/I were larger in the epithelia incubated in the semi-defined media, the I_sc_ responses were smaller; this observation indicates that differences due to incubation in the specific medias go beyond the levels of spontaneous CFTR activity.

**Figure 6:**
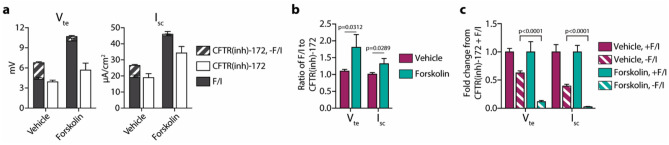
24-h forskolin treatment decreases spontaneous CFTR activity in non-CF epithelia. Epithelia were cultured with either vehicle alone or 10 µM forskolin for 24 h prior to analysis. Epithelia were analyzed in an Ussing chamber using the same methods listed in Fig. 1. (**a**) Responses to forskolin/IBMX (F/I) and CFTR(inh)-172 in the presence or absence of F/I were quantified. Significant differences due to chronic forskolin were detected for F/I responses [*p* < 0.0001 for transepithelial potential difference/voltage (V_te_), *p* = 0.0001 for short-circuit current (I_sc_)], post-F/I CFTR(inh)-172 responses (*p* = 0.0460 for V_te_, *p* = 0.0045 for I_sc_), and spontaneous CFTR activity (*p* < 0.0001 for V_te_ and I_sc_). (**b**) For acutely activated epithelia, the ratio of the change in response to F/I to the change in response to the following CFTR(inh)-172 treatment were calculated. Values shown are absolute. (**c**) Responses to 172 were normalized to the values obtained in epithelia treated with F/I prior to inhibition. n = 1 donor with 3 replicates per condition.

The effect of forskolin addition to the media was also explored. It was previously observed that airway epithelia that underwent ~ 24-h cAMP stimulation during culture had decreased CFTR-mediated anion conductance at baseline^20^. By measuring spontaneous CFTR activity, the decrease in CFTR activity in forskolin-treated epithelia was verified (Fig. 6). While not explored in the present study, this effect is potentially due to a compensatory mechanism in the epithelia that inhibits the unintended (and, consequently, the intended/spontaneous) activation of CFTR channels under the chronic forskolin condition. Interestingly, the forskolin-treated epithelia also demonstrated an increased response to acute F/I.

## Discussion

Overall, the experiments described herein provide evidence of spontaneously active CFTR in nasal airway epithelial cell cultures differentiated at the air–liquid interface in vitro. The levels of spontaneous CFTR activity can be measured by inhibiting CFTR in the absence of CFTR-activating compounds during electrophysiological measurements. When CFTR-activating compounds are employed, the response to subsequent inhibition of CFTR provides a measure of the maximum functional capacity of CFTR in the epithelia (i.e., response to CFTR activation + spontaneous CFTR activity = response to CFTR inhibition after activation = maximum functional capacity of CFTR). Estimation of spontaneous CFTR activity may be made by calculating the difference between electrical parameters before CFTR activation and after stabilization following CFTR inhibition^14,17^, although significant differences between changes obtained through this method and the analysis of spontaneous CFTR activity using CFTR(inh)-172 responses in the absence of F/I were observed (Figs. 1e and S1c).

The magnitude of responses to CFTR modulation in nasal epithelia derived from individuals with CF were highly dependent on the method utilized to measure these responses (Figs. 2, 3, 4). Examples that highlight this observation are the F508del/F508del CFTR epithelial response to VX-809/VX-770 (6.01-fold increase in F/I-induced I_sc_ changes and only 1.49-fold increase in spontaneous CFTR activity, Fig. 2), F508del/R117H CFTR epithelia response to VX-770 (0.98-fold increase in F/I-induced I_sc_ changes and 1.79-fold increase in spontaneous CFTR activity, Fig. 3), and the F508del/F508del CFTR epithelia response to VX-661 alone (9.06-fold increase in F/I-induced I_sc_ changes and only 1.17-fold increase in spontaneous CFTR activity, Fig. 4). While future research should determine which measurement provides a better correlation with in vivo clinical responses to CFTR modulators, the experiments performed in this study demonstrate clear differences between the results obtained using these two distinct methods.

The maximum functional capacity of CFTR that is measured during the use of F/I is distinct from the levels of spontaneous CFTR activity in these epithelia, and we suggest that analyzing changes in the spontaneous CFTR activity in response to CFTR modulation may be valuable in the development of the next generation of CFTR modulators. Importantly, nasal potential difference assays demonstrate that the CFTR present in the upper airway in vivo are not transporting ions at maximal capacity; addition of a chloride gradient and subsequent isoproterenol result in increases in potential, indicating increases in CFTR-mediated chloride transport^21,22^. We suggest that this observation supports the use of spontaneous CFTR activity as a physiologically relevant measure. In addition to F/I-induced changes and spontaneous CFTR activity measurements, it is important to note that, for F508del/F50del CFTR epithelia treated with the VX-445/VX-661/VX-770 triple modulator combination, baseline electrophysiological properties and responses to amiloride were also greatly different from vehicle-treated cells, while treatment with other modulators/combinations did not significantly affect these values (Figs. 2b and S1). This is relevant to manifestations of dysfunctional CFTR in vivo, as hyperpolarization and increased amiloride responses are evident in individuals with CF^20,23^. Therefore, these parameters may also serve as physiologically relevant readouts of in vitro CFTR modulator responses.

VX-770 was included in 24-h CFTR modulator treatments of this study, as there is increased physiological relevance for using this method over only exposing cells to VX-770 acutely during electrophysiological analyses. As mentioned above, while VX-661 treatment alone resulted in no increase in spontaneous CFTR activity in F508del/F508del CFTR epithelia, the addition of VX-770 to VX-661 did result in an increase. This highlights the important concept that increasing the expression of a CFTR mutant that is non-functional has no detectable consequences in these epithelia. The acute exposure of cultures to VX-770 and other potentiators during analysis should be sufficient for initial identification of compounds of interest during drug screens; however, further verification using long-term exposure models is important for the development of these compounds. While the physiological concentrations of bioactive VX-770 and the impact of chronic VX-770 exposure at these concentrations is unclear^19,24–27^, future studies examining the concentration-dependent impact of chronic VX-770 on CFTR using spontaneous measurements may be valuable.

An important caveat of this study is the limited number of unique biological replicates on which experiments were performed. While all experiments utilized rigorous internal controls, the degree of variability in spontaneous CFTR activity and changes in responses to modulator exposures in donor-derived nasal epithelia needs to be defined in future studies with epithelia derived from a larger number of donor samples. This study provides a robust proof of concept for the use of spontaneous CFTR activity as a unique measure of in vitro CFTR modulator responses.

Our findings demonstrate that primary nasal epithelial cell cultures possess CFTR that are actively transporting anions through endogenously regulated mechanisms in the absence of exogenous stimulation. Many factors not examined in this study may influence the endogenous pathways regulating spontaneous CFTR activity, including time of culture at the air–liquid interface, passage number of the cells, and plating density/time of expansion in submerged culture. Additionally, in all experiments reported in this manuscript, epithelia were analyzed under symmetrical chloride conditions; we expect that the presence of a chloride gradient will increase spontaneous CFTR activity. In utilizing airway epithelial cultures, the goal is to accurately represent the airway tissues present in a living individual, providing a physiologically relevant system for studies that cannot be performed in vivo. Reproducing the levels of CFTR activity that are naturally present in the airways is complicated, given the regulation of activity by factors such as beta-adrenergic signaling^28^, VIP^29^, mechanical stress^30^ and others that are absent for our culture system; future studies will aim to optimize culture conditions to provide an accurate representation of in vivo airways, particularly (for CF research) regarding spontaneous CFTR activity. Although the level of spontaneous CFTR activity present in epithelial cells cultured in the conditions described in this study may not accurately reflect the levels of activity in vivo, we suggest that the analysis of spontaneous CFTR activity through the use of CFTR inhibition in the absence of CFTR activation may be more representative of in vivo activity than the levels measured during acute cAMP-mediated activation and subsequent inhibition.

## Methods

### Primary human nasal epithelial cell culture

Primary human nasal epithelial cells were obtained by brushing the inferior turbinate of individuals with and without CF using a nylon cytology brush (Medical Packaging Corporation). This protocol is approved by the National Jewish Health Institutional Review Board (HS-2832) and all donors provided written informed consent prior to brushing. Brushings were processed and expanded according to previously described methods^31–34^. All experiments were performed in accordance with National Jewish Health guidelines and regulations. Cells obtained during brushings were resuspended in conditional reprogramming culture (CRC) medium^32^ supplemented with the ROCK inhibitor Y-27632 (ApexBIO) and plated on an irradiated NIH/3T3 fibroblast feeder layer. Cultures were maintained at 37 °C and 5% CO_2_ in a humidified incubator. Expanded epithelial cells were passaged 2–4 times*,* separated from the fibroblast feeder layer using two-step trypsinization, then plated at 250,000 cells/cm^2^ on 0.4 μm pore size Transwell inserts (Corning) coated with a 3 mg/mL Type I bovine collagen solution (Advanced BioMatrix) and grown submerged in PneumaCult-Ex Plus medium (STEMCELL Technologies) for 40–52 h*.* For differentiation at the air–liquid interface (ALI), apical medium was then removed, and basolateral medium was replaced with PneumaCult ALI Maintenance media (STEMCELL Technologies) for 3–4 weeks to achieve a well-differentiated state^31,33–35^. For a single cell line, epithelia were differentiated at ALI for 3 weeks followed by and additional 96 h in either PneumaCult ALI Maintenance media or a semi-defined media^36^ prior to analysis in the Ussing chamber. For CFTR modulator treatments, 0.1 µM VX-770, 3 µM VX-809, 3 µM VX-661, and/or 3 µM VX-445 diluted in culture medium was added basolaterally to cells for 24 h prior to analysis in the Ussing chamber. Vehicle control treatment consisted of culture medium containing 0.1% DMSO alone.

### Electrophysiological analysis of cultured nasal epithelial cells

Electrophysiological analyses were performed in an Ussing Chamber (Physiologic Instruments, San Diego, CA). Nasal epithelial cell monolayers on inserts were mounted in an Ussing chamber and bathed in a modified Ringer’s solution (120 mM NaCl, 10 mM D-Glucose, 3.3 mM KH_2_PO_4_, 0.83 mM K_2_HPO_4_, 1.2 mM MgCl_2_, 1.2 mM CaCl_2_, 25 mM NaHCO_3_, pH 7.4), maintained at 37ºC and gassed with 5% CO_2_/95% O_2_. Open-circuit conditions with intermittent short-circuiting and voltage pulsing (200 ms pulses at +/− 5 mV) were utilized. With the use of intermittent short circuiting/pulsing, peak values may not have been captured for current and conductance, and values of “0” were utilized when changes in the expected direction were not captured. Cultures were treated acutely in the Ussing chamber with subsequent additions of apical amiloride (100 µM, Alfa Aesar), apical and basolateral forskolin (20 µM, Tocris) and IBMX (100 µM, Sigma) (F/I), apical VX-770 (1 µM, Selleck Chemicals), apical CFTR(inh)-172 (10 µM, CFTR Chemical Compound Distribution Program) and apical ATP (100 µM, Sigma).

### Statistical analyses

Statistical analyses were performed using GraphPad Prism for Mac OS X Version 6.0a. Distributions of values were examined using D’Agostino-Pearson omnibus normality test (Figs. 1, 2), or when group sizes of less than 8 were present (Figs. 3, 4, 5, 6), the Kolmogorov–Smirnov test was utilized. Parametric tests (unpaired t-test and one-way ANOVA with Tukey’s multiple comparisons test) were applied in conditions in which data was normally distributed, and nonparametric tests (Mann–Whitney test) were applied if data failed to pass the normality test described above. For the data shown in Figs. 3, 4, 5 and 6, normality was tested for baseline values and values of change due to amiloride exposure; if the data in each group was found to be normally distributed, then parametric tests were applied to values obtained during post-amiloride treatments.

The results of statistical analyses of selected comparisons are reported. In certain instances, significances are denoted using symbols as follows: “*” for *p* ≤ 0.05, “**” for *p* ≤ 0.01, “***” for *p* ≤ 0.001, “****”*p* ≤ 0.0001, “^#^” for *p* ≤ 0.05 compared to all other groups, and “n.s.” for comparisons that are not statistically significant. For comparisons of the magnitudes of drug responses, values/changes in values of drug-treated epithelia were normalized to mean values of vehicle-treated epithelia in a donor-specific manner. All values shown are means and all error bars are standard deviations.

## Supplementary Information

Supplementary Figures.

## Data Availability

All data relevant to this study are included in this published article and its Supplementary Information files.
