# Supplementary material for: Measurements of spontaneous CFTR-mediated ion transport without acute channel activation in airway epithelial cultures after modulator exposure
Source: Sci Rep. 2021 Nov 19;11:22616. doi: 10.1038/s41598-021-02044-1 (PMC8605007; doi:10.1038/s41598-021-02044-1)
Supplement: Supplementary file 1 — Supplementary Figures. [file 41598_2021_2044_MOESM1_ESM.docx]

**Supplementary Information:**


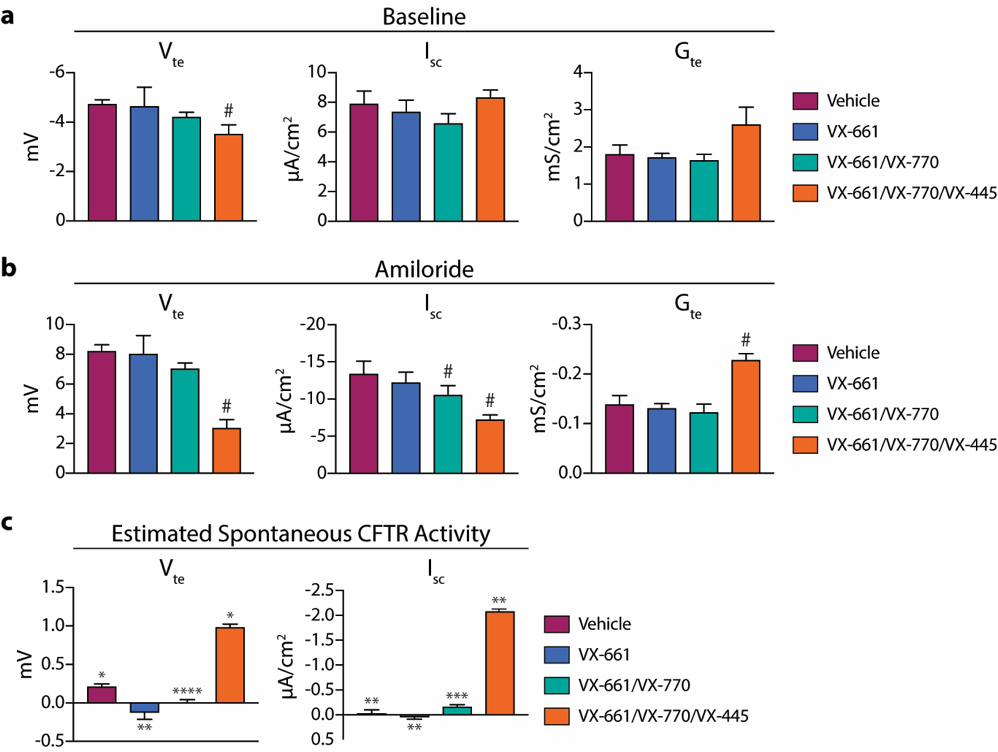


**Fig. S1**. Downstream consequences of increased spontaneous CFTR function and estimated spontaneous CFTR activity in modulator-treated F508del/F508del CFTR epithelia. Changes in baseline measurements (**a**) and response to amiloride (**b**) from the experiment depicted in Figure 4 were quantified. Number signs denote that the indicated group is significantly different from all other groups. (**c**) Estimates of spontaneous CFTR activity were calculated. Asterisks denote differences for the indicated group in the estimated values of spontaneous CFTR-mediated change and the values obtained for the same parameter (-F/I) shown in Figure 4c. n = 3 replicates per condition.


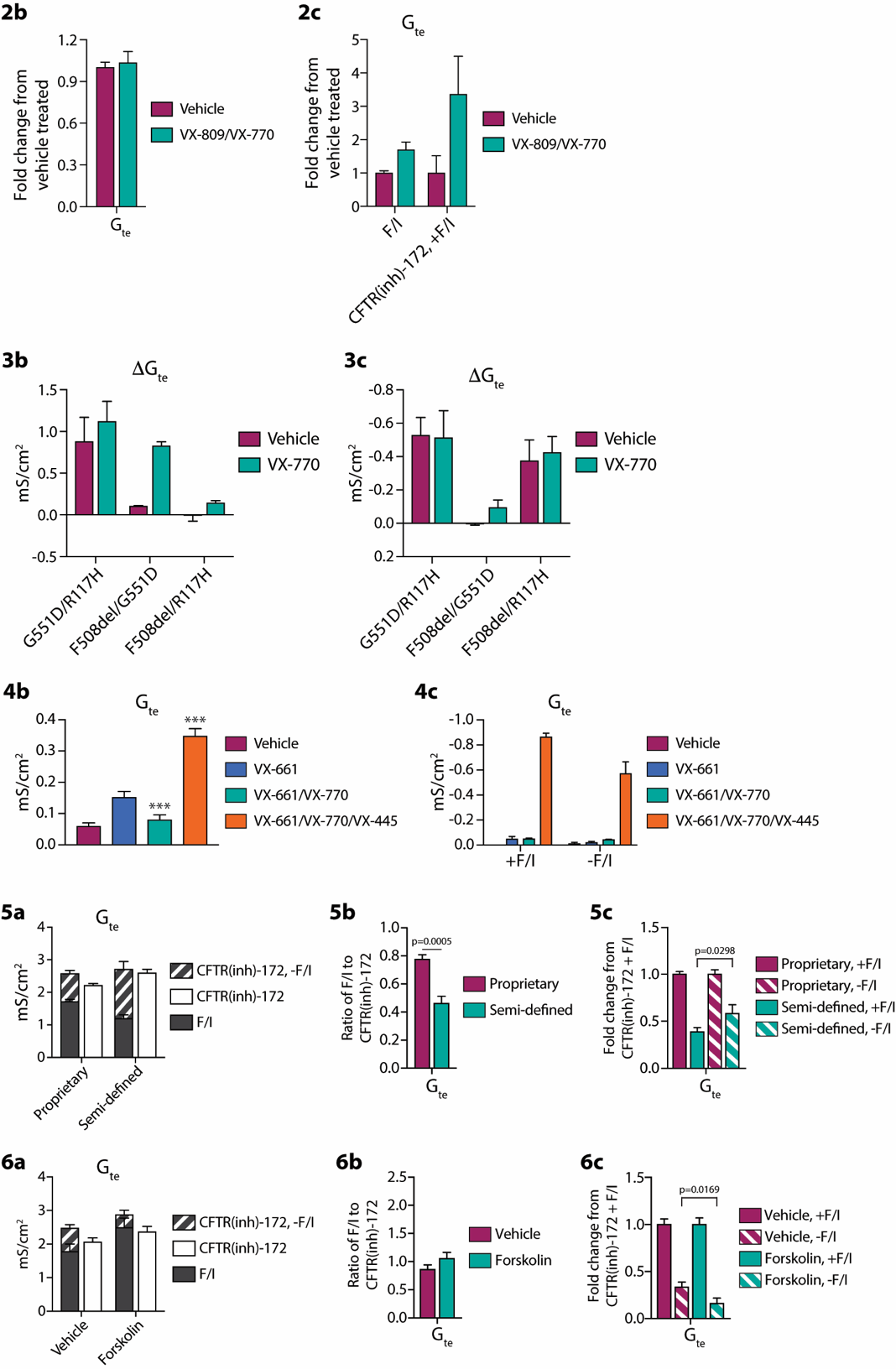


**Fig. S2**. Conductance values obtained during experiments depicted in Figures 2 – 6. Panels are labeled according to the figure panel that displays the corresponding transepithelial potential difference/voltage (V_te_) and short-circuit current (I_sc_) values in the main text. In panels 4b and 4c, asterisks denote differences in the fold increase over vehicle-treated epithelia for the indicated group as compared to the spontaneous CFTR-mediated change for the same group. (**5a**) Significant differences due to media were detected for F/I responses (p=0.0026), post-F/I CFTR(inh)-172 responses (p=0.0079), and spontaneous CFTR activity (p=0.0118). (**6a**) Significant differences due to chronic forskolin were detected for F/I responses (p=0.0272) and spontaneous CFTR activity (p=0.0343).
